# Comparative genetics of *Enterococcus faecalis* intestinal tissue isolates before and after surgery in a rat model of colon anastomosis

**DOI:** 10.1371/journal.pone.0232165

**Published:** 2020-04-28

**Authors:** Scott Christley, Benjamin Shogan, Zoe Levine, Hyun Koo, Kristina Guyton, Sarah Owens, Jack Gilbert, Olga Zaborina, John C. Alverdy

**Affiliations:** 1 Department of Surgery, University of Chicago, Chicago, IL, United States of America; 2 Argonne National Laboratory, Argonne, IL, United States of America; University of Kansas, UNITED STATES

## Abstract

We have recently demonstrated that collagenolytic *Enterococcus faecalis* plays a key and causative role in the pathogenesis of anastomotic leak, an uncommon but potentially lethal complication characterized by disruption of the intestinal wound following segmental removal of the colon (resection) and its reconnection (anastomosis). Here we hypothesized that comparative genetic analysis of *E*. *faecalis* isolates present at the anastomotic wound site before and after surgery would shed insight into the mechanisms by which collagenolytic strains are selected for and predominate at sites of anastomotic disruption. Whole genome optical mapping of four pairs of isolates from rat colonic tissue obtained following surgical resection (herein named “pre-op” isolates) and then 6 days later from the anastomotic site (herein named “post-op” isolates) demonstrated that the isolates with higher collagenolytic activity formed a distinct cluster. In order to perform analysis at a deeper level, a single pair of *E*. *faecalis* isolates (16A pre-op and 16A post-op) was selected for whole genome sequencing and assembled using a hybrid assembly algorithm. Comparative genomics demonstrated absence of multiple gene clusters, notably a pathogenicity island in the post-op isolate. No differences were found in the *fsr-gelE-sprE* genes (EF1817-1822) responsible for regulation and production of collagenolytic activity. Analysis of unique genes among the 16A pre-op and post-op isolates revealed the predominance of transporter systems-related genes in the pre-op isolate and phage-related and hydrolytic enzyme-encoding genes in the post-op isolate. Despite genetic differences observed between pre-op and post-op isolates, the precise genetic determinants responsible for their differential expression of collagenolytic activity remains unknown.

## Introduction

The intestinal tract harbors the most abundant biomass of microbiota in the human body which can vary in both composition and function depending on various conditions including diet, antibiotic use, global travel and physiologic stress [1–4]. Intestinal surgery involves a major stress and disruption of the normal ecology of the microbiota as purgative cleansing is used prior to surgery when antibiotics are administered both orally and parenterally, and the normal anaerobic environment is opened to atmospheric level oxygen. Under such conditions, the relatively low abundance species such as *E*. *faecalis* and *P*. *aeruginosa* become predominant at the sites of the intestinal wound connection or “anastomosis” [5–9]. We have previously demonstrated that such strains play a key and causative role in anastomotic disruption (i.e., leak). [10–12].

Given that *E*. *faecalis* is a low abundance commensal organism comprising less than 1% of the adult gut microflora [13, 14], why its population significantly (up to 500 fold) increases at the site of colon surgery [15] and why isolates express an increased production of collagenolytic activity at these site compared to preoperative isolates [10, 16] is unknown. Here we hypothesized that comparing the genetics of *E*. *faecalis* isolates before and after surgery from the site of the surgical wound would clarify the genes that are involved in the pathogenesis of collagenase-mediated anastomotic leak, as we had demonstrated in a rat model of anastomotic leak induced by *Pseudomonas aeruginosa* [12]. Specifically, a single nucleotide polymorphism (SNP) mutation in the *mexT* gene was found to be responsible for the enhanced collagenolytic activity, with higher incidence of colonies with the SNP mutation at anastomotic sites as compared to other sites of colon [12].

Here we performed genetic analyses of 8 paired isolates of *E*. *faecalis* from the distal colon of rats at the time of colon surgery (4 pre-op isolates) and then 6 days after at the site of the anastomosis (4 post-op isolates). The aim of this study was to determine if there are genetic differences between pre-op and post-op commensal *E*. *faecalis* isolates that explain differences in collagenase activity and their colonization preference for anastomotic tissue sites. Whole genome optical maps of the 8 isolates, and whole genome sequence assembly and annotation of one selected pair, are presented.

## Methods

The animal studies were performed in strict accordance with the recommendations in the Guide for the Care and Use of Laboratory Animals of the National Institutes of Health. The protocol was approved by the institutional animal care and use committee at University of Chicago (IACUC) that as well serves as the animal ethics committee. The Protocol Number: 72085. All surgery was performed under ketamine/xylazine anesthesia, and all efforts were made to minimize suffering.

### Isolation of paired *E*. *faecalis* colonies

The *E*. *faecalis* colonies were isolated from colonic tissue swabs in previously described experiments of rodent colorectal anastomosis model [10] performed in accordance with the guidelines of the institutional animal care and use committee at University of Chicago (ACUP 72085). The rat colon tissue was swabbed at two different time points: at the time of surgery (here named as pre-op) from the resected colon segment, and on the postoperative day 6 from the anastomotic tissue when the rats were sacrificed (here named as post-op). Swabs were plated on Enterococcal selective plates and *Enterococcus* identification was performed by the Vitek 2 system. The isolates were designated with the numbers 12, 16, and 19, reflecting the rat identifications from the original study. From rats 12 and 19, one pair of isolates (pre-op and post-op) from each rat were presented. From rat 16, two pre-op and two post-op isolates were derived. In total, 8 isolates representing 4 pairs of isolates from 3 rats with anastomoses were subjected to genomic analysis.

### Collagenolytic activity

Collagenolytic activity of *E*. *faecalis* isolates was measured as previously described [10] using an EnzChek Gelatinase/Collagenase Assay Kits (Molecular Probes).

### Genome sequence and assembly

Genomic DNA sequencing was performed using the Illumina MiSeq platform. De novo assembly with short-read sequencing tends to produce thousands of contigs, and we felt a higher-quality assembly could be produced with a hybrid approach that combined *de novo* assembly, reference-guided assembly, and whole genome optical mapping. De novo assembly of a total of 26,776,396 high-quality paired-end reads for 16A pre-op and 21,115,709 high-quality paired-end reads for 16A post-op, 101 bp in length, was performed using Spades version 3.5.0 [17], resulting in 9,584 contigs and 5,631 contigs respectively. Reads were aligned to the genomes of seven *E*. *faecalis* strains (V583, Symbioflor1, OG1RF, DENG1, ATCC29212, D32, 62), using Bowtie2 version 2.2.5 [18]. The greatest read coverage was obtained using the V583 genome, which we used for reference-guided assembly. Contigs from *de novo* assembly, greater than 1000bp in length, were placed onto *E*. *faecalis* V583 using BLAST version 2.2.30 [19] and merged into longer scaffolds with reference-aligned consensus sequence using custom Perl scripts. Scaffolds were aligned to the whole genome optical maps using MapSolver^™^ software to verify the position and orientation of the scaffolds. The draft genome sequence of *E*. *faecalis* 16A pre-op is 2,842,456 bp in length across 8 scaffolds (GenBank: LMBS01000000, SRA: SRX1388100), and the draft genome sequence of *E*. *faecalis* 16A post-op is 2,810,200 bp in length across 8 scaffolds (GenBank: LMBT01000000, SRA: SRX1388528). The assembled genomes and sequence read data is available at NCBI under BioProject PRJNA300267. The genomes were annotated using the NCBI Prokaryotic Genome Annotation Pipeline [20], and that annotation was the basis for the comparative genomic analysis. The genomes were also uploaded and analyzed using the Bacterial Analysis Platform [21] which indicated both isolates as sequence type ST-71. We attempted to identify CRISPR elements and *E*. *faecalis* plasmids, but the results were not conclusive; thus, either or both elements may be present in the strains.

### Whole genome optical mapping

High molecular weight genomic DNA for each reference microbe was prepared directly from isolated colonies using the OpGen HMW DNA Isolation Kit. In brief, cells were lysed using OpGen lysis buffer and the lysate diluted for direct use. To reduce DNA shearing, wide-bore pipette tips were used, and DNA-containing solutions were not vortexed. Whole Genome Maps were produced using the ARGUS® Whole Genome Mapping System (OpGen Inc., Gaithersburg, MD). The optimal restriction enzyme NcoI for *E*. *faecalis* was chosen using a software program called Enzyme Chooser (OpGen Inc.) that identifies enzymes that result in a 6–12 kb average fragment size and no single restriction fragment larger than 80 kb across the genome. Single genomic DNA molecules were captured onto an ARGUS surface within a MapCard, digested with a restriction enzyme and stained with JOJO^™^-1 on the ARGUS MapCard Processor, and analyzed by automated fluorescent microscopy using the ARGUS Whole Genome Mapper. Software records the size and order of restriction fragments for each DNA molecule. Collections of single molecule restriction maps are then assembled according to overlapping fragment patterns to produce a Whole Genome Map Assembly. The consensus of the Whole Genome Map Assembly is represented as a Whole Genome Map, where vertical lines indicate the locations of restriction sites and the white space between lines as the size of the restriction fragments in kilobases.

### Genome assembly alignment using MapSolver^TM^

FASTA files were imported into MapSolver^™^ software and converted into in silico maps using the same restriction enzyme. DNA sequence contigs were aligned to the Whole Genome Maps using the sequence placement function of MapSolver, which uses a dynamic programming algorithm that finds the optimal alignment of two restriction maps according to a scoring model that incorporates fragment sizing errors, false and missing cuts, and missing small fragments.

### QRT-PCR analysis of *gelE* expression

*E*. *faecalis* 16A pre-op and 16A post-op isolates were cultured from frozen stocks on tryptic soy broth (TSB) plates and incubated overnight at 37°C. Colonies from TSB plates were suspended in liquid TY medium (10 g/L tryptone and 5 g/L yeast extract) and incubated for 14 hrs at 37°C under static conditions. To note, TY medium is not an optimal medium for *E*. *faecalis* growth; however, specific collagenolytic activity (normalized to growth) is significantly higher on this medium as compared to nutrient rich TSB or Todd Hewitt Broth (Acumedia) (personal observation). The bacterial culture was mixed with two volumes of RNA Protect Bacteria Reagent (Qiagen, Valencia, CA, USA) and incubated for 5 min. Lysozyme (100 μl of 15mg/ml) and proteinase K (10 μl of 600 mAU/ml) were added followed by 10 min incubation at room temperature. RNA was extracted with TRIzol reagent (Invitrogen, Carlsbad, CA) and subsequent purification using RNeasy Plus spin columns (Qiagen, Valencia, CA, USA). DNase treatment was performed using Turbo DNA-free kit (Qiagen, Germany). 1 μg of RNA was used for cDNA synthesis with BioRad iScript Reverse Transcription Supermix (BioRad, Hercules, CA, USA). Quantitative RT-PCR was performed on a QuantStudio^™^ 3 System (Thermo Fisher Scientific) using Sybr Green assays. The program had an initial polymerase activation and the denaturation step at 95°C for 20 sec, and then followed by 40 cycles of 95°C for 1 sec and 60°C for 20 sec. The primer sequences of *gelE* and housekeeping *23S* ribosomal gene were as follows: *gelE* forward primer: 5′-CGG AAC ATA CTG CCG GTT TAG A-3′ and *gelE* reverse primer: 5′-TGG ATT AGA TGC ACC CGA AAT-3′; *23S* forward primer: 5′-CCT ATC GGC CTC GGC TTA G -3′ and *23S* reverse primer: 5′-AGC GAA AGA CAG GTG AGA ATC C-3′. Each sample was run in triplicate, and mean values were used in calculations. The ΔΔCt was used to measure the fold change of *gelE* expression in post-op isolate compared to pre-op isolate. Statistical analysis was performed by Student’s t-test, with p < 0.05 accepted as a statistically significant value. The data were reproduced in two independent experiments.

### Growth curves of *E*. *faecalis* E1 and E2 in TY medium

To characterize the growth of E1 and E2 strains in TY medium, the conditions used for gene expression analysis were used. Exactly, *E*. *faecalis* solution in TY medium prepared from TSB plate colonies contained 11 ml of bacterial solution at OD600nm~0.1. The 15 culture tubes were prepared for each strain, and incubated at 37°C under static conditions. For each strain, 3 tubes were withdrawn at 3, 6, 9, 12, and 24 hrs time points to measure absorbance at OD 600 nm.

## Results

### Whole genome optical mapping reveals distinct clustering of *E*. *faecalis* in association with their collagenolytic activity

**Fig 1:**
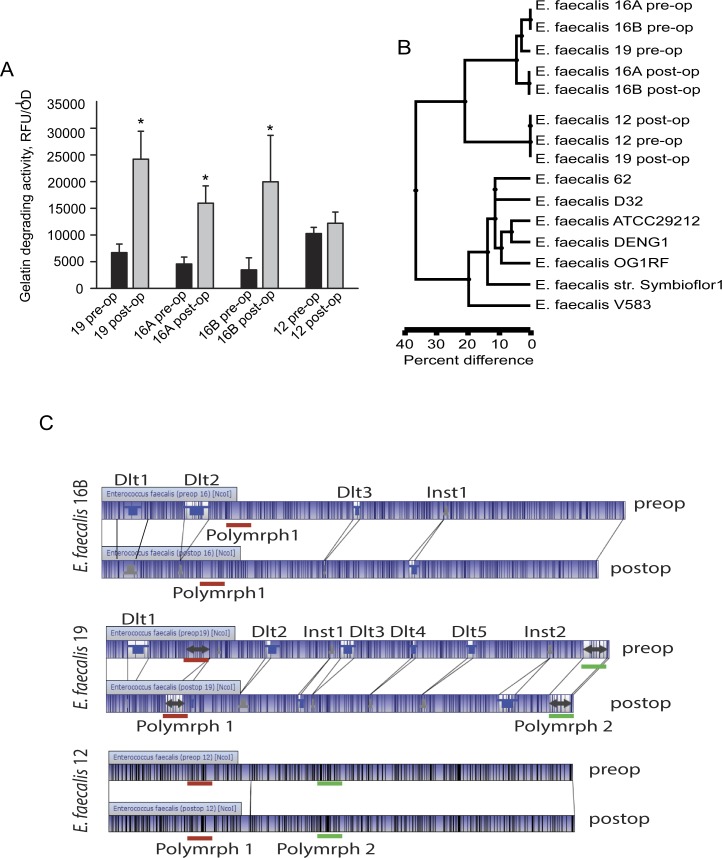
Whole genome optical mapping of *E*. *faecalis* isolated from pre- and post-operative colonic tissues revealed distinct clustering of isolates with higher collagenolytic activity. (**A**), Collagenolytic activity of *E*. *faecalis* isolates based on the fluorescein-labeled gelatin degradation. n = 3, *p<0.01, by Student’s t-test. (**B**), Map similarity clusters based on the whole genome optical mapping. Optical mapping in silico sequences of reference strains was performed based on their whole genome sequences available from the GenBank database. (**C**), Optical maps. Blue-shaded regions between maps represent similar restriction pattern across the chromosome where vertical lines indicate the locations of restriction sites. Blue upside-down top hats represent insertions (Inst) and inverted grey features represent deletions (Dlt). Arrowed area represent polymorphic regions (Polymrph).

We observed that 3 of 4 preoperative isolates displayed lower collagenolytic activity (≤10,000 RFU/OD) compared to the higher collagenolytic activity of post-operative isolates (>10,000 RFU/OD) (**Fig 1A**), as measured by their capacity to degrade fluorescein labeled gelatin. Both pre-op and post-op isolates from rat 12 displayed similar collagenolytic activity of around 10,000 RFU/OD. It is important to note that despite relative differences in collagenolytic activity, there were no isolates that completely lost the ability to degrade gelatin. A dendrogram of whole genome optical mapping (**Fig 1B**) revealed three distinct clusters: I.) 16A pre-op, 16B pre-op and 19 pre-op, with the lower collagenolytic isolates; II.) 16A post-op and 16B post-op with higher collagenolytic activity; and III.) distant cluster of 12 post-op, 12 pre-op, and 19 post-op isolates with higher collagenolytic activity. All rat *E*. *faecalis* isolates clustered separately from reference strains (**Fig 1B**). Detailed analysis of optical mapping revealed no major changes in genomes of the pre- and post-op isolates from rat 12. Additionally, no major differences were found between the pairs isolated from the same rat (number 16) (**S1 Fig**). However, there are numerous differences between pre-op and post-op isolates (**Fig 1C**) that included multiple deletions, insertions, and polymorphic regions. Notable was a dramatic reduction in the genome size of the post-op 16A, 16B, and 19 (by 165 kb in 16A, 16B) and (225 kb in 19). Given numerous deletions, insertions and polymorphic regions between genomes in the lower collagenolytic activity pre-op isolates and in the higher collagenolytic activity post-op isolates, it can be inferred that they represent different strains among the commensal *E*. *faecalis* population. For example, the initial pre-op *E*. *faecalis* population may be represented by both low and high collagenolytic strains (isolate 12 pre-op is an example).

### Whole genome sequence of E. faecalis 16A pre-op and 16A post-op revealed multiple differences yet they are nearly identical in the fsr-gelE-sprE region responsible for the regulation and production of collagenase activity

We first confirmed that the expression of *gelE* was higher in the 16A post-op as compared to 16A pre-op isolate. *gelE* expression was measured and normalized to the housekeeping 23S ribosomal gene in 16A pre-op and 16A post-op isolates grown on TY medium for 14 hrs. Growth characteristics were similar for these strains (**S2A Fig**), however there was a ~7 fold higher expression of *gelE* in 16A post-op as compared to 16A pre-op (**S2B Fig**). These data confirmed previous results demonstrating enhanced collagenolytic phenotypic activity in the 16A post-op isolate as compared to the pre-op isolate.

**Fig 2:**
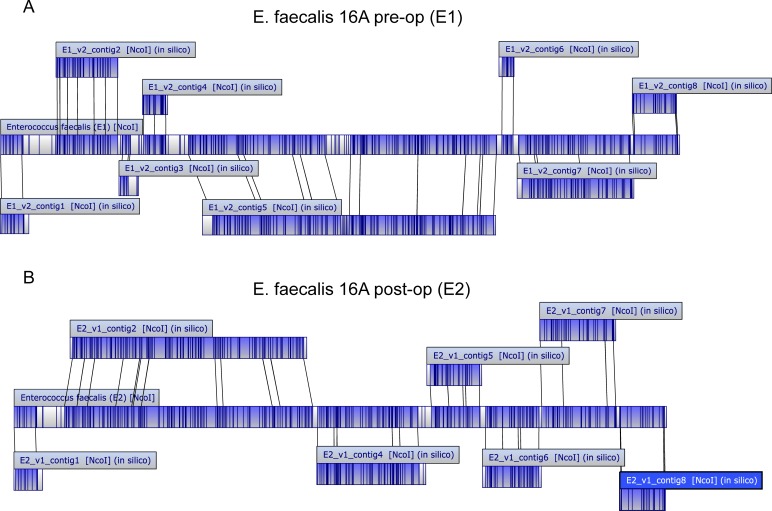
Alignment of scaffolds to optical maps. (**A,B**), alignment of the 16A pre-op isolate (**A**) and the 16A post-op isolate (**B**). The center long rectangle is the optical map as given by OpGen, and each of the smaller long rectangles represents assembled scaffolds. It shows how each scaffold lines up positionally (and in correct orientation) with the optical map.

In order to perform an in-depth head-to-head analysis of a representative pre-op and post-op isolates, we chose 16A pre-op and 16A post-op. These isolates were designated as *E*. *faecalis* E1 and E2 respectively in a recent publication [10]. Whole genome sequencing of these isolates was performed using the Illumina MiSeq platform. A hybrid approach was used for genome assembly by combining alignment to the *E*. *faecalis* V583 genome and *de novo* assembly. For verification, the assembled contigs were aligned to whole genome optical maps using the sequence placement function of MapSolver. The position and orientation of the assembled contigs aligned well to the optical maps covering 2,842,456 bp of the expected genome size of the 16A pre-op isolate (**Fig 2A**) and 2,810,200 bp of the expected genome size of the 16A post-op isolate (**Fig 2B**).

**Fig 3:**
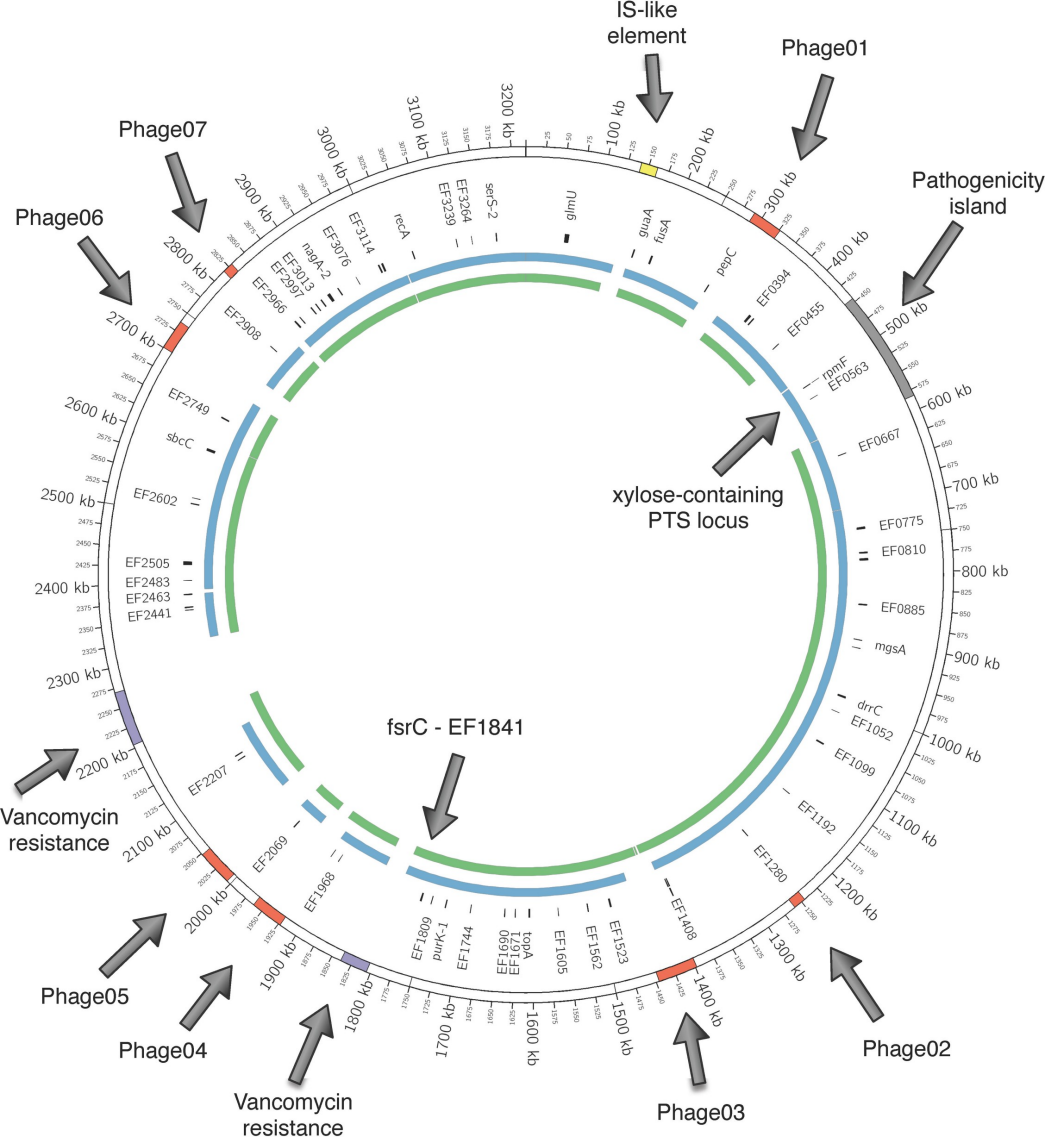
16A pre-op and post-op genomes aligned to the V583 genome. The outer ring represents the 3.2 Mbp genome of V583 with specific regions annotated including the IS-like element (yellow), the seven phage elements (orange), the pathogenicity island (grey), and the two vancomycin resistance islands (purple). The inner rings represent the 16A pre-op isolate (blue) and 16A post-op isolate (green) genomes aligned to the V583 genome. Genes shared by 16A pre-op and 16A post-op, which have variant protein coding, are annotated with their position on the 16A pre-op genome.

Comparative genomics was performed for the 16A pre-op, 16A post-op, and *E*. *faecalis* V583 reference genome (**Fig 3**). 16A pre-op and 16A post-op genomes were annotated using the NCBI Prokaryotic Genome Annotation Pipeline producing 2764 coding sequences including 75 RNAs for 16A pre-op and 2752 coding sequences including 77 RNAs for 16A post-op. 2607 genes are shared between the isolates genomes with 76 of them having non-synonymous mutations (**S1 Table**). 16A pre-op has 157 unique genes and 16A post-op has 145 unique genes. Both isolates lack the vancomycin resistance encoded genes EF1955-EF1963 and EF1869-EF1863 [22] that are present in the V583 genome. Both 16A pre-op and 16A post-op isolates lack an IS-like element suggesting that these isolates are compatible with other commensal enterococci [23]. 16A pre-op shares the pathogenicity island (PAI) [24] in common with V583 but only one of the seven phage elements in V583 are present in this isolate. The 16A post-op lacks the PAI which accounts for the majority of the size reduction in its genome. The majority of unique genes in 16A pre-op as compared to 16A post-op corresponds to the pathogenicity island (PAI) [24, 25].

Among the 157 genes unique to 16A pre-op, 57 are annotated as hypothetical, and 23 encode transporter proteins including 11 phosphotransferase system (PTS) -related genes (**S2 Table**). Among the 145 genes unique to 16A post-op, 95 are annotated as hypothetical, 20 are phage-related, 3 facilitate transcription and replication of the genome including DNA replicase, DNA topoisomerase, and recombinase, and 7 are hydrolytic enzymes such as MBL fold metallo-hydrolase, 3 are peptidases, one is N-acetylmuramoyl-L-alanine amidase, and one is 1,4- beta-N-acetylmuramidase. There are also 2 transporter-encoding genes (**S3 Table**).

We next set out to analyze the *fsr-gelE-sprE* genomic region (EF1817—EF1822) known to be responsible for the production of gelatinase and collagenolytic activity [26, 27]. This analysis demonstrated that 16A pre-op and 16A post-op isolates are identical in this regard (**S4 Table**). We then extended this analysis to the *EF1814—EF1841* region, as it has been previously shown that strains lacking this region do not produce gelatinase [28]. This region was also nearly identical between pre-op and post-op isolates with only a single gene (EF1823) that was different (**S5 Table)**. S5 Table provides the sequence alignment for EF1823, and the alignment shows the introduction of a 6 amino acid insertion and a single amino acid insertion near the beginning of the gene. The putative product of EF1823 showed sequence similarities to some N-acetyl muramidases [29] with potential autolytic activity [30]. GelE is known to be involved in the maturation of the *E*. *faecalis* muramidase [31], and GelE-producing strains have increased autolysis [32, 33]; however, the involvement of EF1823 in the regulation of *gelE-sprE* expression is not known.

## Discussion

Although a key and causative role for collagenolytic *E*. *faecalis* in the pathogenesis of anastomotic leak has been confirmed in several reports [5, 10, 16, 34], the genetic basis by which preoperative strains at anastomotic sites differ in terms of their collagenolytic potential and colonization preference compared to those present on the anastomotic wound site several days later remains elusive. Given the numerous deletions, insertions and polymorphic regions between genomes in lower collagenolytic potential pre-op isolates and higher collagenolytic post-op isolates, it can be inferred that isolates represent distinct populations colonizing colonic tissues during surgery vs many days following surgery when present on a healing anastomotic wound. The high rate of colonization by collagenolytic strains that we previously observed on healing anastomotic wound several days after surgery [10, 35, 36] suggests that the healing anastomotic environment is a preferable niche for collagenolytic strains. Initial strains present on normal colonic tissue might represent a population of *E*. *faecalis* with varying levels of collagenolytic activity. Environmental factors at the healing anastomotic tissue site on the other hand may harbor “cues” that select for strains with higher collagenolytic activity. Among such cues might be factors present in the exposed extracellular matrix (ECM) that are not present when tissues are intact. These could include but not be limited to fibrinogen and fibronectin [37–39] in which bacterial adhesion is enhanced in the presence of serum [40], another common exposure as a result of surgical injury to the colon at a anastomotic site. The GelE gelatinase of *E*. *faecalis* responsible for its collagenolytic activity is a broad spectrum protease with the ability to cleave fibrins [32, 38, 41]. *E*. *faecalis* uses fibrinogen to support its growth [39], requiring collagenolytic activity to break down the nascent molecule. Further work using metabolomics and proteomics will be required to fully elucidate the local cues present in a healing anastomotic wound that select for *E*. *faecalis* and activate its adherence and expression of collagenase.

Finally, results from the present study beg the question of how *gelE* and the level of collagenolytic activity between the 16A pre-op and post-op strains can occur without obvious genetic differences in the *fsr-gelE-sprE* region. The genetic determinants that regulate the level of expression of collagenase are not known. We do not suspect one is related to the loss of PAI since the V583 strain that harbors PAI expresses high collagenolytic activity. More likely is the possibility that phage-related genes enriched in post-op strain may contribute to the enhanced expression of *gelE* through epigenetic regulation. From another site, it is important to test if the EF1823 contributes to the changes in the expression of *gelE*. Further work will be required to identify which genetic determinants are responsible for the observed enhanced collagenolytic activity in *E*. *faecalis* harvested from anastomotic wound that might require an assessment of not only its *in vivo* expression, but how strains evolve over the course of the treatment from the pressures of colonic surgery.

### Ethics statement

The animal studies were performed in strict accordance with the recommendations in the Guide for the Care and Use of Laboratory Animals of the National Institutes of Health. The protocol was approved by the institutional animal care and use committee at University of Chicago (IACUC) that as well serves as the animal ethics committee. The Protocol Number: 72085. All surgery was performed under ketamine/xylazine anesthesia, and all efforts were made to minimize suffering.

## Supporting information

S1 Table 16A pre-op and post-op shared genes with non-synonymous mutations. (XLSX)

S2 Table Genes unique to 16A pre-op compared to 16A post-op genome. (XLSX)

S3 Table Genes unique to 16A post-op compared to 16A pre-op genome. (XLSX)

S4 Table Comparison of EF1814-EF1841 regions of 16A pre-op and 16A post-op genomes. (XLSX)

S5 Table Alignment of EF1823 gene in pre-op and post-op strains. (DOCX)

S1 Fig Optical maps of 16A pre-op, 16A post-op, 16B pre-op and 16B post-op isolates. Blue-shaded regions between maps represent similar restriction pattern across the chromosome where vertical lines indicate the locations of restriction sites. Blue upside-down top represent insertions (Inst) and inverted grey features represent deletions (Dlt). Arrowed area represent polymorphic regions (Polymrph).
(PDF)

S2 Fig Higher level of *gelE* expression in the 16A post-op isolate is observed at the similar growth characteristics of pre-op and post-op isolates in TY medium. **(A),** Growth of 16A pre-op (black circles) and post-op (grey circles) in TY medium. N = 3, data are represented by average values, arrow represent standard deviations. Arrow with small values are not visible. **(B),** Relative expression of *gelE* at 14 hrs in 16A post-op compared to 16A pre-op isolate. Results represent mean of two independent experiments. *P<0.05 by Student t-test.
(DOCX)
