# Supplementary material for: Comparative genetics of Enterococcus faecalis intestinal tissue isolates before and after surgery in a rat model of colon anastomosis
Source: PLoS One. 2020 Apr 28;15(4):e0232165. doi: 10.1371/journal.pone.0232165 (PMC7188289; doi:10.1371/journal.pone.0232165)
Supplement: S5 Table — (DOCX) [file pone.0232165.s005.docx]

Match [First Match](https://blast.ncbi.nlm.nih.gov/Blast.cgi#hspQuery_28533_1)

| Alignment statistics for match #1 | | | | | | |
| --- | --- | --- | --- | --- | --- | --- |
| **Score** | **Expect** | **Method** | **Identities** | **Positives** | **Gaps** | **Frame** |
| 1309 bits(3387) | 0.0() | Compositional matrix adjust. | 649/656(99%) | 649/656(98%) | 7/656(1%) |  |

Features:

Preop 16 MKRKKIKNQLLVLTSILALLSLEVAPVVTLAAELPSSSLQTALSSETTITSEEKVI---- 56

MKRKKIKNQLLVLTSILALLSLEVAPVVTLAAELPSSSLQTALSSETTITSEEKVI

Postop16 MKRKKIKNQLLVLTSILALLSLEVAPVVTLAAELPSSSLQTALSSETTITSEEKVIKTTE 60

Preop 57 --ETTVATSTTSTSSSSSESNSSTDTTTESTSHSTAETTTTNTSSETKKEPTESTVSSEI 114

ETTVATSTTSTSSSSSESNSSTDTTTESTSHSTAETTTTNTSSETKKEPTESTVSSEI

Postop 61 TTETTVATSTTSTSSSSSESNSSTDTTTESTSHSTAETTTTNTSSETKKEPTESTVSSEI 120

Preop 115 TQPVEQSQPTQV-PVTKQEPEEPIQVPEATNNVVEENQAVSLNPSLKVDEIASSNLKGYE 173

TQPVEQSQPTQV PVTKQEPEEPIQVPEATNNVVEENQAVSLNPSLKVDEIASSNLKGYE

Postop 121 TQPVEQSQPTQVQPVTKQEPEEPIQVPEATNNVVEENQAVSLNPSLKVDEIASSNLKGYE 180

Preop 174 LPLLSSFGEKKRAVVVAEALRHVGKTKKEFNLTEQALTSSFLAQKIYQQLFKIDIGSTPQ 233

LPLLSSFGEKKRAVVVAEALRHVGKTKKEFNLTEQALTSSFLAQKIYQQLFKIDIGSTPQ

Postop 181 LPLLSSFGEKKRAVVVAEALRHVGKTKKEFNLTEQALTSSFLAQKIYQQLFKIDIGSTPQ 240

Preop 234 EQITFGKVRSIEEAEPGDLIFWQTAEGKTLQNGVYLGQGKYLIAAAEADSKEEPEVIGQL 293

EQITFGKVRSIEEAEPGDLIFWQTAEGKTLQNGVYLGQGKYLIAAAEADSKEEPEVIGQL

Postop 241 EQITFGKVRSIEEAEPGDLIFWQTAEGKTLQNGVYLGQGKYLIAAAEADSKEEPEVIGQL 300

Preop 294 ENIYTAKQQPDSKEGKRLVVTNPFKEFTLTEYGKEVLATYGASFEMQKSEQTTAFIKKIG 353

ENIYTAKQQPDSKEGKRLVVTNPFKEFTLTEYGKEVLATYGASFEMQKSEQTTAFIKKIG

Postop 301 ENIYTAKQQPDSKEGKRLVVTNPFKEFTLTEYGKEVLATYGASFEMQKSEQTTAFIKKIG 360

Preop 354 ETARELGEKYDVFASVMIAQAILESGSGESQLAKEPYYNLFGVRGSFQGNSVSFSTKEAD 413

ETARELGEKYDVFASVMIAQAILESGSGESQLAKEPYYNLFGVRGSFQGNSVSFSTKEAD

Postop 361 ETARELGEKYDVFASVMIAQAILESGSGESQLAKEPYYNLFGVRGSFQGNSVSFSTKEAD 420

Preop 414 QRGQLYTISAGFRDYGGYNDSLQDYVQLLRQGIDGNQDFYKPAWRSEAKNYLQATRFLTG 473

QRGQLYTISAGFRDYGGYNDSLQDYVQLLRQGIDGNQDFYKPAWRSEAKNYLQATRFLTG

Postop 421 QRGQLYTISAGFRDYGGYNDSLQDYVQLLRQGIDGNQDFYKPAWRSEAKNYLQATRFLTG 480

Preop 474 KYATDKQYDNKLNSLIAVYNLTQFDLPKTVDGLIIQSKNKLSEAEQQQMHFPVYDGINYN 533

KYATDKQYDNKLNSLIAVYNLTQFDLPKTVDGLIIQSKNKLSEAEQQQMHFPVYDGINYN

Postop 481 KYATDKQYDNKLNSLIAVYNLTQFDLPKTVDGLIIQSKNKLSEAEQQQMHFPVYDGINYN 540

Preop 534 RSGSYPVGQCTWYVYNRFKQLGTSVDEFMGNGSDWGRKGRALGYQVSSLPKAGRAISFQP 593

RSGSYPVGQCTWYVYNRFKQLGTSVDEFMGNGSDWGRKGRALGYQVSSLPKAGRAISFQP

Postop 541 RSGSYPVGQCTWYVYNRFKQLGTSVDEFMGNGSDWGRKGRALGYQVSSLPKAGRAISFQP 600

Preop 594 GVAGADNQYGHVAFVEAVTSDGIIISESNVINDQTISYRVLPNVIAYSSGVTYIGA 649

GVAGADNQYGHVAFVEAVTSDGIIISESNVINDQTISYRVLPNVIAYSSGVTYIGA

Postop 601 GVAGADNQYGHVAFVEAVTSDGIIISESNVINDQTISYRVLPNVIAYSSGVTYIGA 656
