# Supplementary figures and images for: Comparative genetics of Enterococcus faecalis intestinal tissue isolates before and after surgery in a rat model of colon anastomosis
Source: PLoS One. 2020 Apr 28;15(4):e0232165. doi: 10.1371/journal.pone.0232165 (PMC7188289; doi:10.1371/journal.pone.0232165)

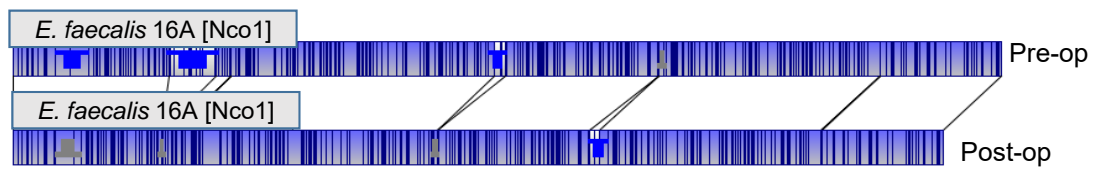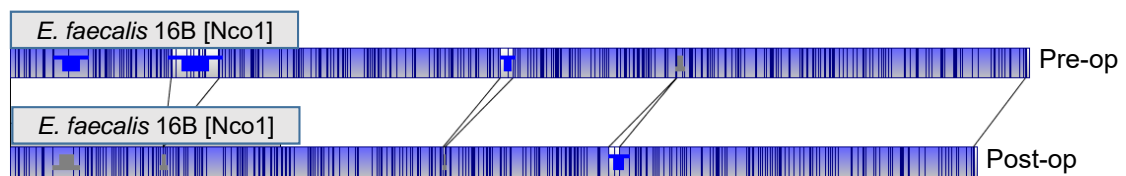

Supplement: S1 Fig — Blue-shaded regions between maps represent similar restriction pattern across the chromosome where vertical lines indicate the locations of restriction sites. Blue upside-down top represent insertions (Inst) and inverted grey features represent deletions (Dlt). Arrowed area represent polymorphic regions (Polymrph). (PDF) [file pone.0232165.s006.pdf]
