# Supplementary material for: Comparative genetics of Enterococcus faecalis intestinal tissue isolates before and after surgery in a rat model of colon anastomosis
Source: PLoS One. 2020 Apr 28;15(4):e0232165. doi: 10.1371/journal.pone.0232165 (PMC7188289; doi:10.1371/journal.pone.0232165)
Supplement: S2 Fig — (A), Growth of 16A pre-op (black circles) and post-op (grey circles) in TY medium. N = 3, data are represented by average values, arrow represent standard deviations. Arrow with small values are not visible. (B), Relative expression of gelE at 14 hrs in 16A post-op compared to 16A pre-op isolate. Results represent mean of two independent experiments. *P<0.05 by Student t-test. (DOCX) [file pone.0232165.s007.docx]

A B

*

**Fig.S2**. **Higher level of *gelE* expression in the 16A post-op isolate is observed at the similar growth characteristics of pre-op and post-op isolates in TY medium**. (**A**), Growth of 16A pre-op (black circles) and post-op (grey circles) in TY medium. N=3, data represented by average values, arrow represent standard deviations. Arrow with small values are not visible. (**B**), Relative expression of *gelE* at 14 hrs in 16A post-op compared to 16A pre-op isolate. Results represent mean of two independent experiments. *P<0.05 by Student t-test.
